# Association between multiple infection patterns of HPV33 and the risk of cervical carcinogenesis

**DOI:** 10.3389/fmicb.2026.1787378

**Published:** 2026-05-25

**Authors:** Wenqian Shi, Wenjie Qu, Yaping Wang, Fang Chen, Zhiheng Wang, Qi Zhou, Tianyi Bi, Pei Zhang, Jingyi Feng, Fangying Chen, Lin Lin, Xing Liu, Yifei Yao, Zhiyong Wu, Yan Wang, Yanyun Li

**Affiliations:** 1 Obstetrics and Gynecology Hospital of Fudan University, Shanghai Key Laboratary of Reproduction and Development, Shanghai Key Laboratary of Female Reproductive Endocrine Related Diseases, Shanghai, China; 2 Department of Epidemiology, School of Public Health, Fudan University, Shanghai, China; 3 Med-X Research Institute, School of Biomedical Engineering, Shanghai Jiao Tong University, Shanghai, China

**Keywords:** cervical intraepithelial lesions, HPV16, HPV33, multiple infection, single infection

## Abstract

**Background:**

Human papillomavirus 33 (HPV33) is among the five most prevalent HPV genotypes in China and is commonly involved in co-infections. However, the synergistic effects of specific genotype combinations on cervical carcinogenesis remain incompletely understood. This study aimed to characterize HPV33 co-infection patterns and their associated genetic variations in relation to cervical lesion progression.

**Methods:**

We enrolled 1,770 HPV33-positive patients from the Obstetrics and Gynecology Hospital of Fudan University between 2018 and 2023, including 852 with HPV33 single infections and 918 with multiple infections. Logistic regression was used to assess associations between co-infection characteristics and cervical histopathological results. In a subset of 90 cases, the full-length L1 gene of HPV33 was sequenced and phylogenetically analyzed to evaluate genomic variation by infection status.

**Results:**

The number of HPV33 co-infecting genotypes was positively correlated with cervical lesion severity (*p* < 0.05). HPV52, HPV16, and HPV58 were the most frequent co-infecting genotypes and were associated with an increased risk of cervical intraepithelial lesion progression: HPV16 (OR 1.97, 95% CI: 1.17–3.34), HPV52 (OR 2.07, 95% CI: 1.26–3.39), HPV58 (OR 2.83, 95% CI: 1.42–5.64). However, among HPV33 multiple infections (≥3 genotypes), only those that involved HPV16 were directly and significantly associated with an increased risk of cervical lesions, with ORs increasing from 1.72 (95% CI: 1.03–2.85) for LSIL to 3.16 (95% CI: 1.75–5.72) for HSIL or worse. Phylogenetic analysis classified most HPV33 sequences into sublineage A1, with no significant difference in L1 gene mutations between single and multiple infections.

**Conclusion:**

HPV33 co-infection patterns, particularly those involving HPV16, are consistently associated with an elevated risk of high-grade cervical lesions in this Chinese cohort. These findings underscore the differential risks associated with distinct HPV33 co-infection patterns and support genotype-specific risk stratification in cervical cancer screening programs.

## 1 Introduction

Cervical cancer is the fourth most common cancer among women worldwide and is the second most common to breast cancer in Asia (Sung et al., 2021). Persistent high-risk human papillomavirus (hrHPV) infection is the primary cause of the development of cervical precancerous lesions and cancer (Shamseddine et al., 2021). So far, more than 400 different HPV genotypes have been found, which have been further classified into low-risk HPV (lrHPV), probable high-risk HPV (phrHPV), and hrHPV based on their distinct carcinogenicity (Arbyn et al., 2014).

Multiple infection is defined as the simultaneous presence of two or more distinct HPV genotypes in the same individual, which is common, ranging from 20% to over 50% among HPV-positive women (Carrillo-García et al., 2014; Zhang et al., 2016; Chen et al., 2022; Zhou et al., 2024). The association between multiple HPV infections and cervical disease progression remains controversial (Song et al., 2022). Several studies have reported that multiple hrHPV infections might contribute to an increased risk of cervical lesions, suggesting potential synergistic effects on cervical carcinogenesis (Cassani et al., 2024a,b; Luo et al., 2024; Su et al., 2024). In contrast, others supported the opposite results that there is no inevitable connection between multiple infections and lesion progression (Tong et al., 2024; Zhong et al., 2024; Baek et al., 2025; Song et al., 2025). These findings suggest an undefined association between HPV co-infections and cervical lesions. Moreover, the factors contributing to this uncertainty remain to be elucidated. One of the major reasons is that most studies do not specifically clarify whether cervical disease risk is driven by the number of co-infecting genotypes or by specific co-infection patterns.

To date, most studies on multiple HPV infections have predominantly focused on HPV16, whereas other genotypes are limited. The prevalence of HPV33 in China ranks in the top five among Chinese women with normal cervical cytology, cervical lesions, and cervical cancer (de Martel et al., 2017). HPV33 is frequently found in co-infections with HPV16, HPV18, and HPV31, although the association with the risk of cervical lesions remains unclear (Gu et al., 2016; Liao et al., 2020; Laake et al., 2022; Luo et al., 2023). An epidemiological study from China has shown that HPV33 ranked second only to HPV16 in cervical intraepithelial neoplasia grade 2 or worse (CIN2+), with its progression risk independent of co-infection status (Song et al., 2022). Another retrospective study showed that HPV33 infection with high viral load, whether single or co-infection, was associated with a higher risk of developing high-grade squamous intraepithelial lesion (HSIL) and cervical cancer (Wang et al., 2020). Moreover, HPV33 lineages and sublineages should also be considered. Compared with other sublineages, sublineage A1 has been linked to a high risk of CIN2+ (Xi et al., 2014; Yan et al., 2023). However, studies focusing on the number and genotype composition of HPV33 multiple infections remain scarce so far.

The present study was conducted to investigate the proportion of individuals with HPV33 single and multiple infections among a large population from the largest cervical disease diagnosis and treatment center in China. It also focused on the multiple infection patterns, which were defined as different combinations of HPV33 co-infected with other HPV genotypes, and evaluated whether these patterns would influence the risk of cervical lesions. The results will complement the limited research on HPV33 infection patterns and their impacts.

## 2 Materials and methods

### 2.1 Study population and design

This study contains two parts. Firstly, we investigate the distribution of HPV33 single and multiple infections, as well as their correlation with the levels of cervical pathological lesions. Secondly, we further explore the relationship between HPV33 genetic diversity and susceptibility to multiple infections.

In the first part, a total of 1,770 patients' pathologic results and personal information were collected from the Cervical Disease Diagnosis and Treatment Center, Obstetrics and Gynecology Hospital of Fudan University. Inclusion criteria were defined as follows: (1) all patients got a cervical exfoliative cytology collection and HPV genotyping test; (2) cervical histological diagnosis within 6 months of genotyping test; (3) HPV positive and HPV genotyping results contain HPV33. The exclusion principles were lack of specific HPV genotyping test and incomplete patient information. Cervical biopsy samples were obtained by physicians qualified for colposcopy examination using biopsy forceps under colposcopic guidance from colposcopically abnormal areas, and fixed in 10% neutral-buffered formalin for histopathological assessment. The histopathological results were classified as normal (including inflammation), low-grade squamous intraepithelial lesion (LSIL), HSIL, and cancer (CA).

In the second part, 147 cervical exfoliative cell samples were collected from November 2020 to August 2023 in the Cervical Disease Diagnosis and Treatment Center, Obstetrics and Gynecology Hospital of Fudan University, and successfully 90 HPV33 L1 full-length sequences were retrieved, including 47 from single infections and 43 from multiple infections, to further analyze the phylogenetics of HPV33 with single and multiple infections. The inclusion criteria included: (1) HPV genotyping results containing HPV33; (2) all patients voluntarily agreed to participate in this study. The exclusion criteria included: (1) incomplete patient information; (2) DNA extraction was unsuccessful. Cervical exfoliated cell samples were collected by two physicians qualified for colposcopy examination using a cytobrush, stored in phosphate-buffered saline (PBS), and then placed on ice for transport to the laboratory. Upon arrival, the samples were aliquoted; half was used for DNA extraction that evening, and the remaining half was stored at −80 °C as a backup.

### 2.2 DNA extraction, amplification, and L1 sequence

Genomic DNA was extracted from the cervical exfoliated cells using the TIANamp Genomic DNA Kit (DP304, TIANGEN, China). To amplify the full-length of the HPV33 L1 gene, the type-specific primers were designed. The sequences of primers used to amplify the full-length of the HPV33 L1 gene were as follows: sense 5′-GGCGTAAACGTTTTCCATATTTT-3′, anti-sense 5′-AACAACAACATAACACAATTACACAA-3′. The specific primers were designed by the NCBI Primer-BLAST tool. Using clinically confirmed HPV33-positive samples as templates, PCR amplification was performed with the synthesized primers. The amplicons were subjected to Sanger sequencing, and the resulting sequences were aligned with the HPV33 L1 gene sequence (GenBank accession No. M12732.1) using BLAST, which showed 100% identity, confirming that the primers specifically amplify the target HPV33 fragment. This primer pair has already been used in related studies (Qu et al., 2024). PCR reactions were conducted in a 50 μl reaction volume containing one unit of TaKaRa Ex Taq (RR001, TaKaRa, Japan), 1 × Ex Taq Buffer (Mg^2+^ plus), 200 μM of dNTP Mixture, and 20 pmol of each primer. The thermal cycling parameters were 98 °C for 10 s, 55 °C for 10 s, 72 °C for 90 s, with 35 cycles and a final extension at 72 each primer. The thermal cycamplicons were sequenced by the ABI 3730xl DNA analyzer. All samples underwent twice PCR amplification and bidirectional sequencing to exclude PCR artifacts.

### 2.3 Phylogenetic analysis and sequence alignment

The Maximum likelihood tree based on the HPV33 L1 genes was conducted in MEGA v11.0 using the Tamura 3-parameter model with 1,000 bootstrap replicates. The HPV33 standard sequences of each lineage and sublineage (A1: M12732; A2: HQ537698; A3: EU918766; B: HQ537705; C: KF436865) were all downloaded from NCBI GenBank database (Burk et al., 2013). All obtained sequences were aligned with HPV33 prototypes by MEGA v11.0 (Tamura et al., 2021). The pairwise comparison was visualized in Microsoft Excel.

### 2.4 Statistical analysis

All statistical analyses were performed using SPSS software (version 26.0) and Graphpad Prism (version 9.5.1). Continuous variables were presented as mean ± standard deviation (SD), while categorical variables were summarized as counts and percentages. Group differences were evaluated using the Pearson chi-square test or Fisher's exact test, with a *p*-value <0.05 regarded as statistically significant. Binary logistic regression was applied to estimate odds ratios (ORs) across groups. For analysis, LSIL+ was defined to include LSIL, HSIL, and CA, whereas HSIL+ was defined to include HSIL and CA. Thus, HSIL+ represents a subset of LSIL+ and was used to assess the risk associated with higher-grade lesions.

### 2.5 Ethics statement

The present study protocol was reviewed and approved by the Institutional Review Board of Obstetrics and Gynecology Hospital of Fudan University (Ethical Approval No. 2025-185). Informed consent forms were waived due to retrospective data analysis reasons in the first part. Informed consent was obtained from all patients included for HPV sequencing in the second part.

## 3 Results

### 3.1 Characteristics of the study population

**Figure 1:**
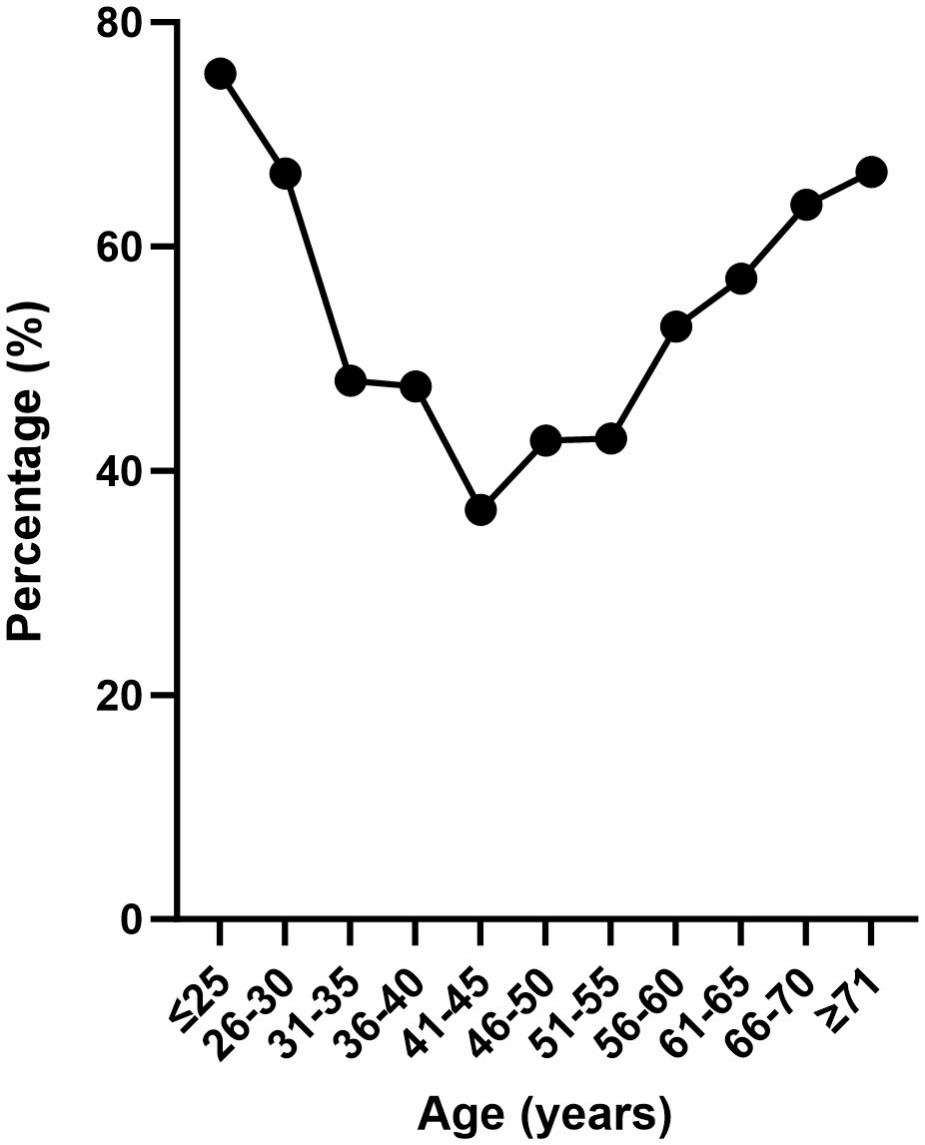
Percentage of the age-specific distribution of HPV33 multiple infections.

**Figure 2:**
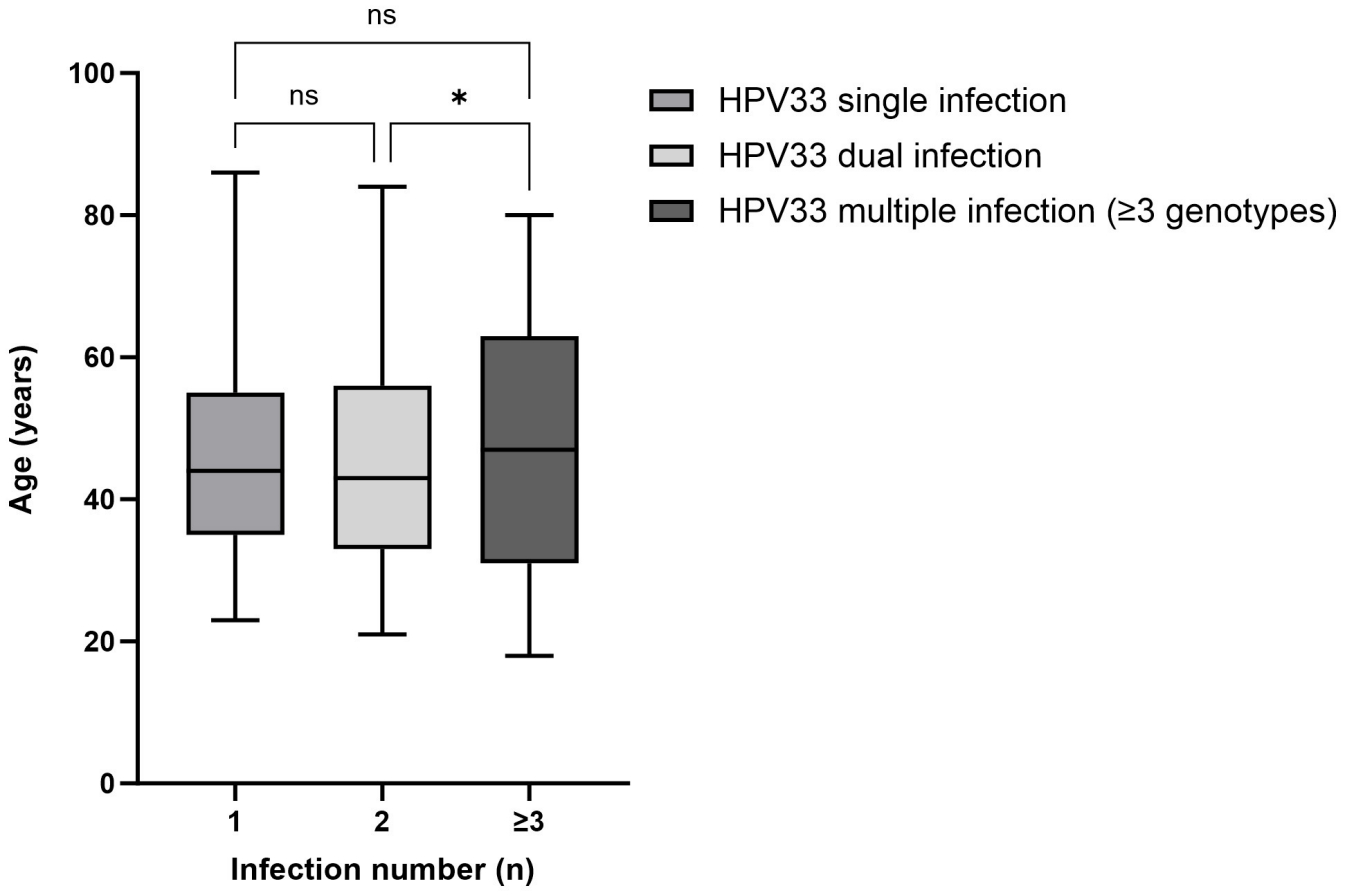
Age distribution of HPV33 single and multiple infections. ns, not significant (*p* > 0.05). *p < 0.05.

A total of 1,770 patients were included in the first part of this study (Supplementary Figure 1A). The baseline characteristics were described in Table 1. Among these patients, 852 (48.14%) were HPV33 single-infected and 918 (51.86%) were co-infected. No significant difference in mean age was observed between HPV33 single infection population and multiple infection population. The age-specific distribution of HPV33 multiple infections exhibited a “U-shaped” pattern, with higher proportions observed in the young and older ages (Figure 1). Individuals infected with more than three HPV genotypes showed a higher median age than those infected with two genotypes (Figure 2). Compared with HPV33 single infections, multiple infections showed a stronger association with pathological outcomes, with the number of co-infecting genotypes ranging from 2 to 11 (*p* < 0.0001). Furthermore, patients were sub-grouped based on the number of co-infecting genotypes to assess their association with the severity of cervical lesions.

**Table 1:** Characteristics of the study population.

Characteristics	Single infection (*n* = 852)	Multiple infection (*n* = 918)	*p-*value
Age, mean ± SD, years	45.94 ± 12.764	45.80 ± 15.107	0.831
Histopathology, *n* (%)			<0.0001
Normal	467 (54.81%)	351 (38.24%)	
LSIL	227 (26.64%)	377 (41.07%)	
HSIL	151 (17.71%)	177 (19.28%)	
CA	7 (0.82%)	13 (1.42%)	
HPV genotype number, *n* (%)			/
1	852 (100%)		
2	/	497 (54.14%)	
3	/	230 (25.05%)	
4	/	112 (12.20%)	
5	/	47 (5.12%)	
6	/	18 (1.96%)	
7	/	4 (0.44%)	
8	/	6 (0.65%)	
9	/	2 (0.22%)	
11	/	2 (0.22%)	
Mixed phrHPV, *n* (%)			/
Yes	/	320 (34.86%)	
No	/	598 (65.14%)	
Mixed lrHPV, *n* (%)			/
Yes	/	165 (17.97%)	
No	/	753 (82.03%)	

### 3.2 Association between the number of HPV33 co-infecting genotypes and cervical lesions

In Table 2, the number of HPV33 co-infections was categorized into three groups (1, 2, and ≥3 genotypes). The group of patients co-infected with more than three genotypes showed a significant association with LSIL+ (*p* < 0.05). In addition, all cases were further categorized into three comparative groups, including HPV33 + hrHPV, HPV33 + phrHPV, and HPV33 + lrHPV, aiming to explore their association with cervical lesions (Normal vs. LSIL+). Compared with single infection, within the HPV33 + hrHPV group, an increasing number of co-infecting genotypes was positively associated with LSIL+ (*p* < 0.0001); however, there was no significant association between the HPV33 + phrHPV/lrHPV group and LSIL+; in HPV33 dual infections, the combination involving hrHPV had a stronger relationship with LSIL+ compared with those involving phrHPV (*p* = 0.0141). Therefore, we further compared different infection patterns in relation to cervical lesion grades.

**Table 2:** Analysis of the number and composition of HPV33 co-infections.

Infection number	HPV33 + hrHPV		*p*-value	HPV33 + phrHPV		*p*-value	HPV33 + lrHPV		*p*-value
	Normal (%)	LSIL+^a^ (%)		Normal (%)	LSIL+ (%)		Normal (%)	LSIL+ (%)	
1	467 (54.81%)	385 (45.19%)	<0.0001	/	/	0.5529	/	/	0.3438
2	129 (38.51%)	206 (61.49%)	0.0343	57 (51.82%)	53 (48.18%)	/	25 (48.08%)	27 (51.92%)	/
≥3	39 (28.26%)	99 (71.74%)		4	6		1	1	

^*a*^LSIL+ includes LSIL, HSIL, and CA.

### 3.3 Association between HPV33 dual infection patterns and the risk of cervical lesions

**Figure 3:**
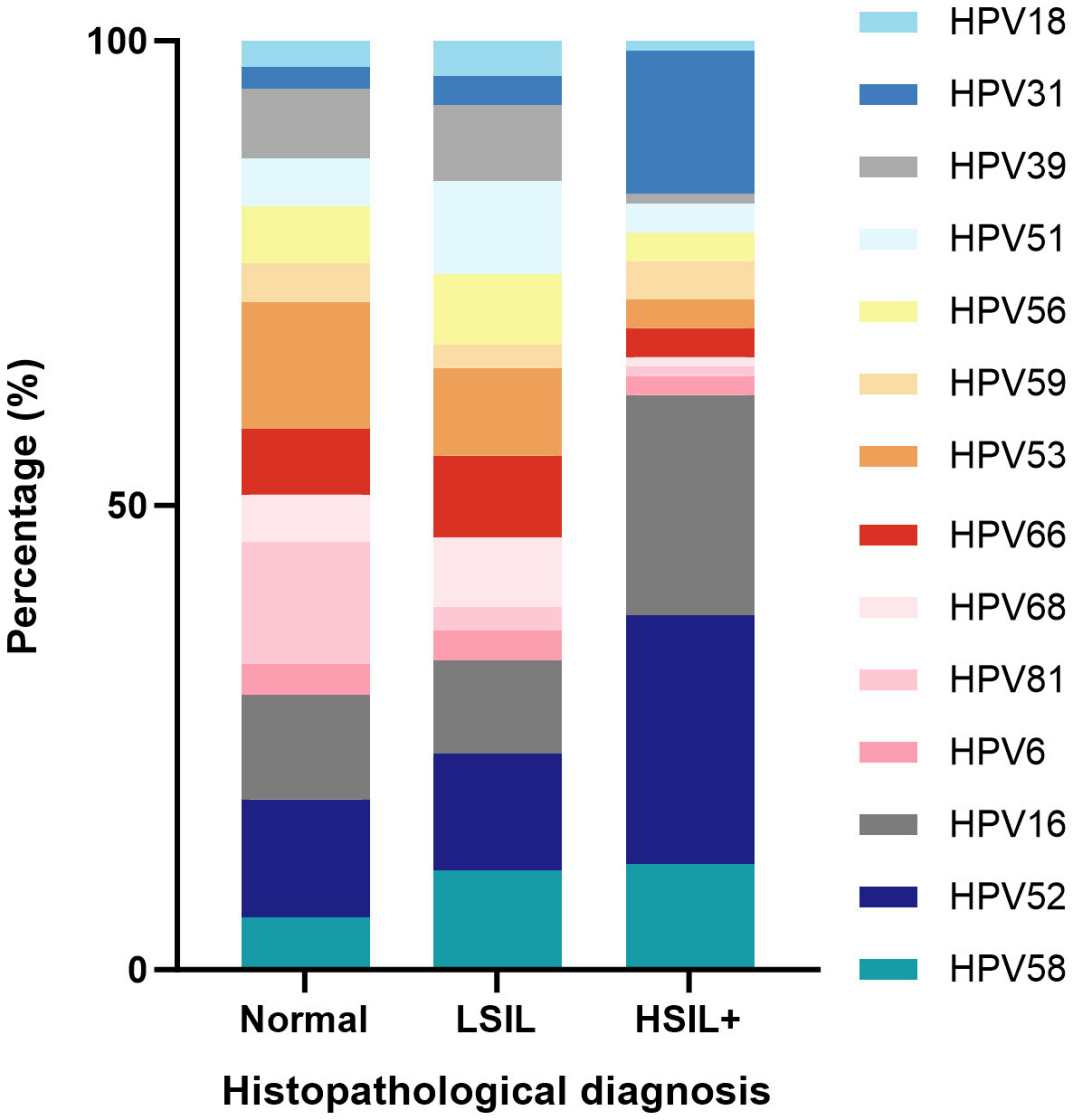
Percentage of 14 HPV33 dual infection patterns across different histopathological grades (Normal, LSIL, HSIL+).

The proportion of each genotype found in co-infection with HPV33 varied notably with the progression of cervical lesions. It was evident that HPV16, HPV52, and HPV58 accounted for the highest proportions across all grades. Moreover, HPV16 (23/97, 23.71%), HPV52 (26/97, 26.80%) and HPV31 (15/97, 15.46%) markedly increased in HSIL+ compared with Normal and LSIL (Figure 3).

**Figure 4:**
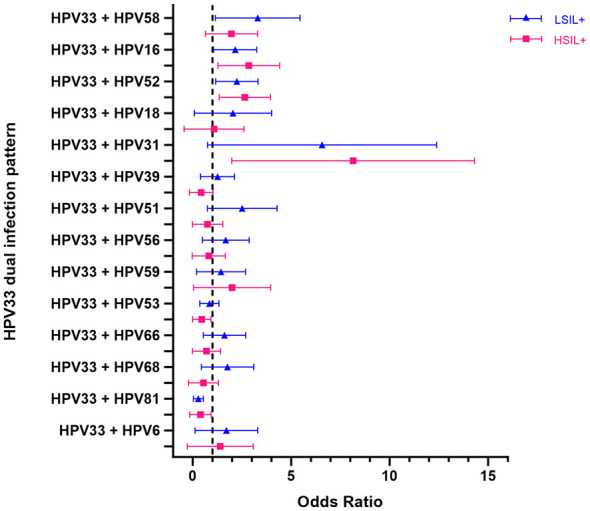
Logistic regression of the risk of cervical lesions of HPV33 dual infections. The vertical dashed line refers to the line of no effect (OR = 1).

Based on these findings, we further compared each combination with HPV33 single infection using binary logistic regression (Table 3, Figure 4). The combination of HPV33 + HPV58 (OR 2.83, 95% CI: 1.42–5.64), HPV33 + HPV16 (OR 1.97, 95% CI: 1.17–3.34), HPV33 + HPV52 (OR 2.07, 95% CI: 1.26–3.39), and HPV33 + HPV31 (OR 4.85, 95% CI: 1.80–13.05) were significantly associated with an increased risk of LSIL+. Moreover, HPV33 + HPV16 (OR 2.53, 95% CI: 1.47–4.56), HPV33 + HPV52 (OR 2.43, 95% CI: 1.46–4.04), and HPV33 + HPV31 (OR 6.59, 95% CI: 2.91–14.94) also increased the risk of HSIL+, except for HPV58. HPV81 (OR 0.22, 95% CI: 0.08–0.57) and HPV53 (OR 0.30, 95% CI: 0.09–0.98) might exert a potential protective effect against cervical lesions.

**Table 3:** The risk of cervical lesions of HPV33 dual infections.

Dual infection pattern	No. of LSIL+	*p*-value	OR (95% CI)	No. of HSIL+^a^	*p-*value	OR (95% CI)
Only HPV33	385		1	158		1
HPV33 + HPV58	28	0.003	2.83 (1.42–5.64)	11	0.162	1.67 (0.82–3.41)
HPV33 + HPV16	39	0.011	1.97 (1.17–3.34)	23	0.001	2.53 (1.47–4.56)
HPV33 + HPV52	46	0.004	2.07 (1.26–3.39)	26	0.001	2.43 (1.46–4.04)
HPV33 + HPV18	7	0.536	1.42 (0.47–4.25)	1	0.336	0.37 (0.08–2.84)
HPV33 + HPV31	20	0.002	4.85 (1.80–13.05)	15	0.000	6.59 (2.91–14.94)
HPV33 + HPV39	14	0.873	1.06 (0.51–2.20)	1	0.064	0.15 (0.02–1.12)
HPV33 + HPV51	19	0.055	2.10 (0.99–4.46)	3	0.243	0.49 (0.15–1.63)
HPV33 + HPV56	15	0.383	1.40 (0.66–2.98)	3	0.300	0.53 (0.16–1.77)
HPV33 + HPV59	8	0.878	1.08 (0.41–1.38)	4	0.603	1.35 (0.44–4.20)
HPV33 + HPV53	18	0.357	0.75 (0.41–1.38)	3	0.046	0.30 (0.09–0.98)
HPV33 + HPV66	17	0.378	1.38 (0.68–2.79)	3	0.198	0.45 (0.14–1.51)
HPV33 + HPV68	13	0.386	1.43 (0.64–3.24)	1	0.106	0.19 (0.03–1.43)
HPV33 + HPV81	5	0.002	0.22 (0.08–0.57)	1	0.051	0.14 (0.02–1.01)
HPV33 + HPV6	7	0.720	1.21 (0.42–3.49)	2	0.685	0.73 (0.16–3.30)

^*a*^HSIL+ includes HSIL and CA; 95%CI, confidence interval.

### 3.4 Association between HPV33 multiple infection (≥3 genotypes) patterns and the risk of cervical lesions

**Figure 5:**
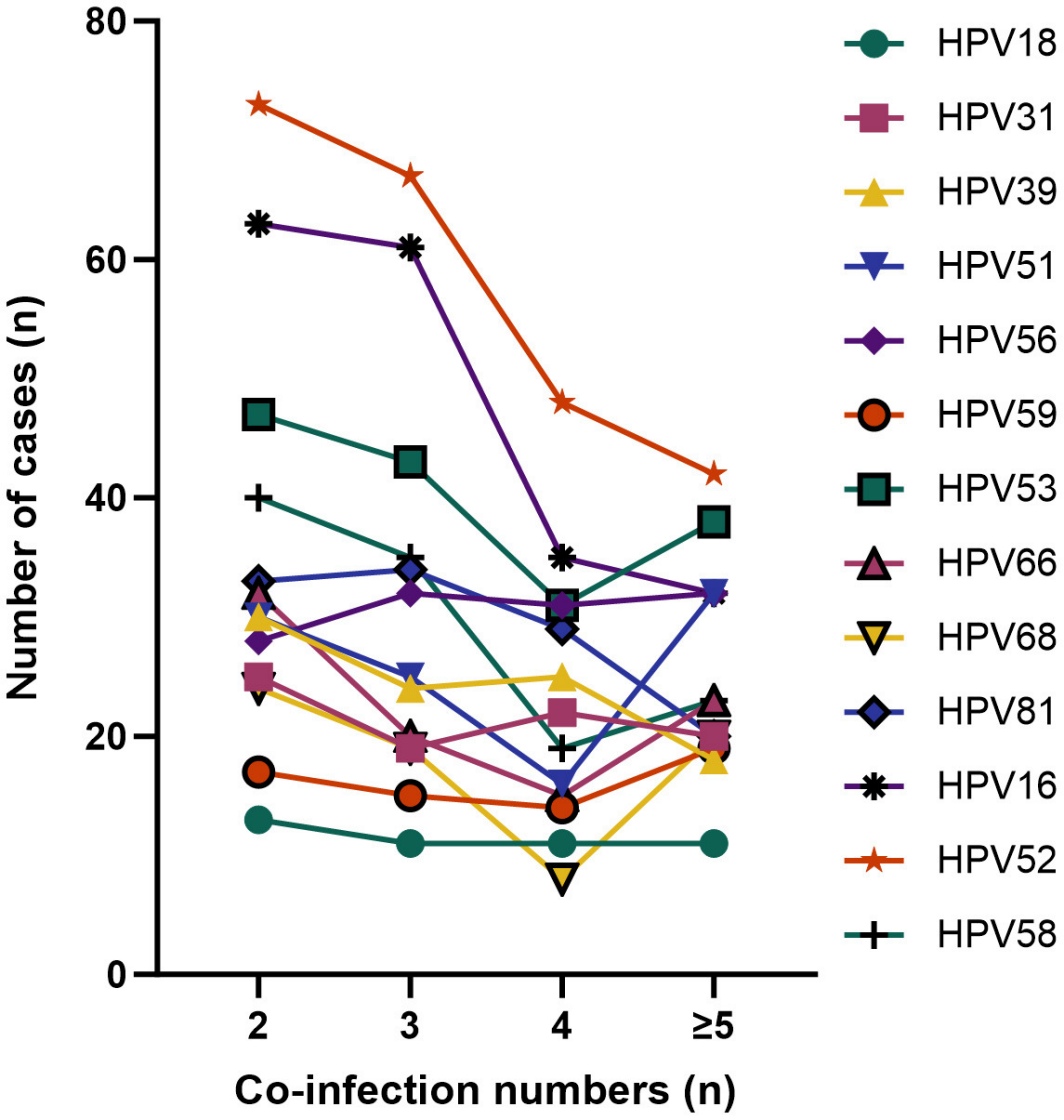
Trends in case numbers of genotypes co-infecting with HPV33 across increasing co-infection group sizes.

Notably, HPV16, HPV52, and HPV58 remained the most frequent partners of HPV33 as the number of co-infecting genotypes increased, whereas other genotypes, such as HPV18, HPV39, and HPV59, were less represented and exhibited relatively stable proportions (Figure 5). Therefore, in HPV33 multiple infections (≥3 genotypes), the associations of HPV16, HPV52, and HPV58 positivity with cervical lesion grades were further evaluated (Table 4). Only HPV16 positivity showed a significant association with LSIL (OR 1.72, 95% CI: 1.03–2.85) and HSIL+ (OR 3.16, 95% CI: 1.75–5.72), whereas HPV52 or HPV58 showed no significant associations.

**Table 4:** Association of HPV33 multiple infection (≥3 genotypes) with or without HPV16, HPV52, and HPV58 with cervical lesions.

Histopathology	+HPV16^a^	–HPV 16^b^	*p-*value	OR (95% CI)	+HPV52^c^	–HPV 52^d^	*p-*value	OR (95% CI)	+HPV58^e^	–HPV 58^f^	*p*-value	OR (95% CI)
Normal	29	111	0.001	1	50	90	0.894	1	28	112	0.699	1
LSIL	61	136	0.037	1.72 (1.03–2.85)	75	122	0.659	1.11 (0.71–1.74)	36	161	0.691	0.89 (0.52–1.55)
HSIL+	38	46	<0.001	3.16 (1.75–5.72)	32	52	0.720	1.11 (0.63–1.94)	13	71	0.398	0.73 (0.36–1.51)

^*a*^HPV33 multiple infections (≥3 genotypes) with HPV16.

^*b*^HPV33 multiple infections (≥3 genotypes) without HPV16.

^*c*^HPV33 multiple infections (≥3 genotypes) with HPV52.

^*d*^HPV33 multiple infections (≥3 genotypes) without HPV52.

^*e*^HPV33 multiple infections (≥3 genotypes) with HPV58.

^*f*^HPV33 multiple infections (≥3 genotypes) without HPV58.

### 3.5 Phylogenetic analysis and lineage/sublineage classification of genetic variations in HPV33 infections

**Figure 6:**
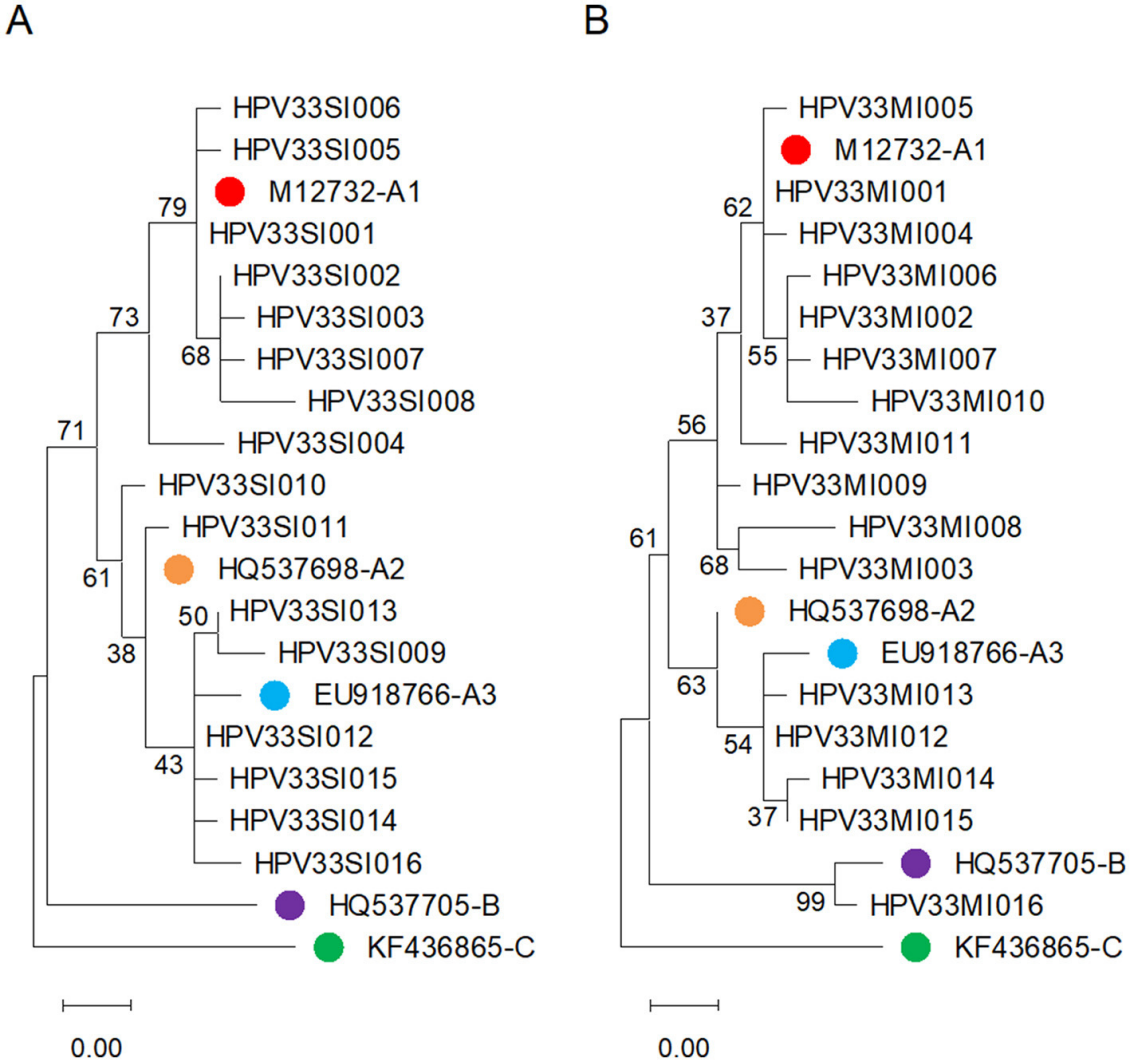
Phylogenetic trees of HPV33 L1 genes from single and multiple infection groups. **(A)** Phylogenetic trees of HPV33 L1 genes from single infection groups. **(B)** Phylogenetic trees of HPV33 L1 genes from multiple infection groups. Reference sequences are highlighted with solid dots.

**Figure 7:**
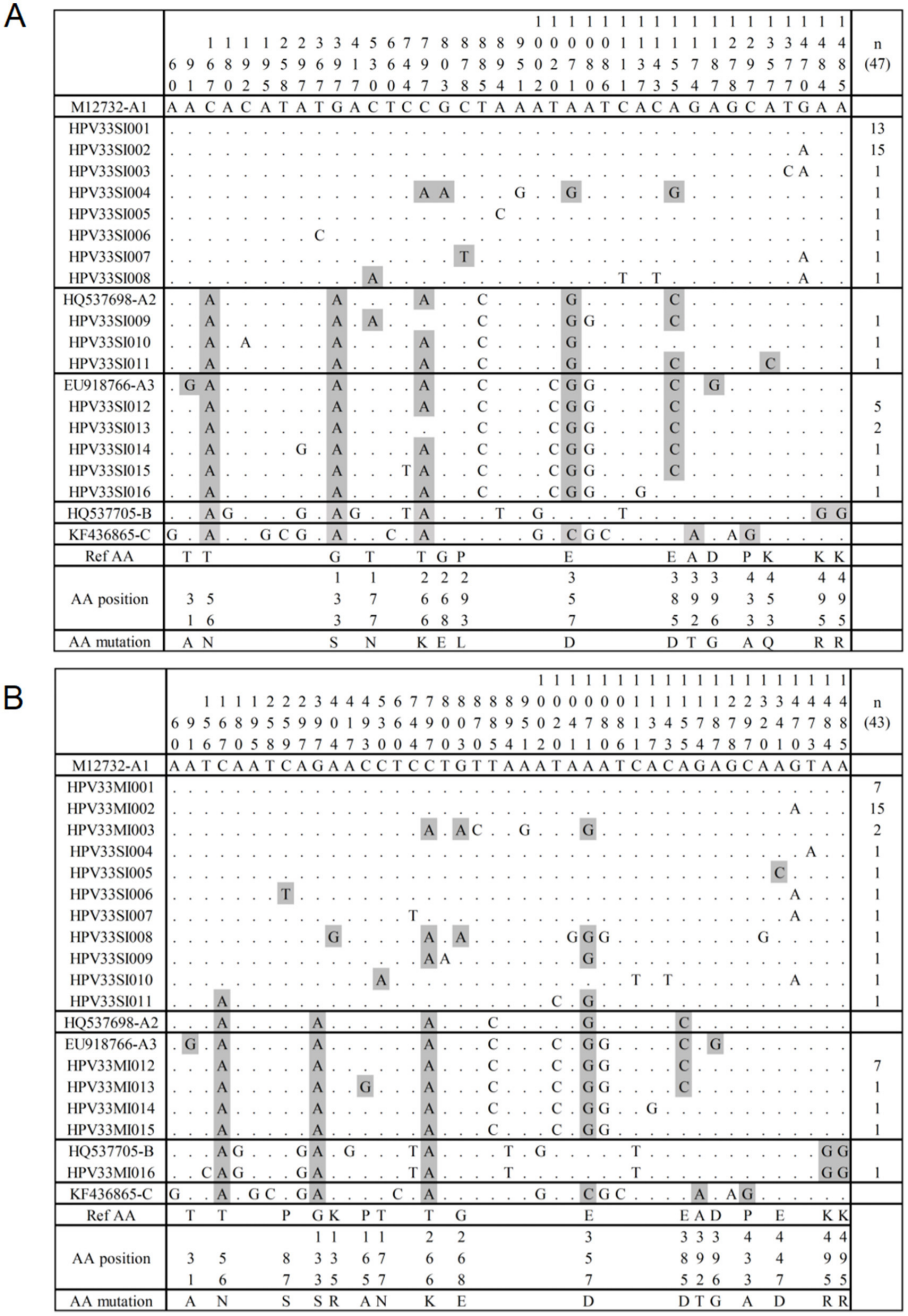
Sequence alignment of HPV33 L1 nucleotide sequences in single and multiple infection groups. **(A)** Sequence alignment of HPV33 L1 nucleotide sequences in single infection groups. **(B)** Sequence alignment of HPV33 L1 nucleotide sequences in multiple infection groups. All sequence numbering is relative to the HPV33 A1 prototype reference (GenBank: M12732). Nucleotide substitutions were marked in gray.

In the second part, we finally collected 47 HPV33 single infection and 43 HPV33 multiple infection samples to further analyze phylogenetics of HPV33 with single and multiple infections (Supplementary Figure 1B). First, we generated maximum likelihood phylogenetic trees for each group (Figure 6). In HPV33 single infection group, 72.34% (34/47) of L1 isolates belonged to A1 sublineage, while 6.38% (3/47) and 21.28% (10/47) belonged to A2 and A3 sublineage, respectively. In HPV33 multiple infection group, 74.42% (32/43) amplified L1 sequences were divided into A1 sublineage, and 23.26% (10/43) were divided into A3 sublineage. Only one isolate was classified into the B lineage. Furthermore, we aligned all the obtained HPV33 L1 sequences with the sublineage A1 standard sequence in both groups (Figure 7). Twenty-nine synonymous and 19 non-synonymous nucleotide substitutions were identified separately. However, no significant difference was observed in the distribution of synonymous and non-synonymous mutations between HPV33 single and multiple infections (*p* > 0.05), nor among each mutated nucleotide site.

## 4 Discussion

The present study investigated the multiple infection patterns of HPV33 and their correlation with cervical lesions within a retrospective analysis based on clinical and laboratory data from 1,770 HPV33-positive patients. Although the number of HPV33 co-infections contributed to the risk of cervical lesions, the infection patterns appeared to play an even more decisive role, especially when hrHPV was involved. In HPV33 dual infection, HPV33 + HPV16, HPV33 + HPV52, HPV33 + HPV58, and HPV33 + HPV31 contributed to the increased risk of cervical intraepithelial lesion progression. However, within HPV33 multiple infections (≥3 genotypes), the significant association with cervical lesion risk remained exclusive in the HPV16-positive group.

There is currently no unified conclusion on the association between multiple infections and cervical lesions. The interactions among different HPV genotypes are complex, involving either synergistic or independent carcinogenic effects on pathogenicity (Trottier et al., 2006; Brant et al., 2020; Luo et al., 2024). While some studies reported that only certain high-risk HPV genotypes may play a dominant role in the development of cervical lesions (Insinga et al., 2008). Kim et al. reported that multiple HPV infections are associated with persistent infections, and they further increased the risk of cervical diseases (Spinillo et al., 2016; Pimenoff et al., 2018; Kim et al., 2021). Here, we concluded that HPV33 multiple infections promoted cervical lesion progression, but it encompassed various aspects. For example, consistent with previous research, our study also demonstrated that an increasing number of HPV33 co-infection with hrHPVs showed a strong association with LSIL+. However, taking the infection number alone into account is incomplete and potentially misleading.

As the study progressed, we found that compared with HPV33 + phrHPV/lrHPV, HPV33 co-infection with hrHPV exhibited a stronger association with LSIL+. This finding provided preliminary evidence that the carcinogenicity of co-infecting genotypes might play a pivotal role in the association with cervical lesions. Therefore, in HPV33 dual infections, co-infection with hrHPV genotypes prevalent in China, including HPV16, HPV52, HPV58, and HPV31, significantly increased the risk of LSIL+, which was in accordance with prior research (Lin et al., 2024; Su et al., 2024). Accordingly, we expanded the scope of infection numbers in our analysis to further confirm the strong oncogenic potential of these hrHPV genotypes.

In HPV33 multiple infections (≥3 genotypes), the presence of HPV16 maintained a strong association with cervical lesions, suggesting that HPV16 retained its dominant oncogenic function even within a heterogeneous viral background. However, the presence or absence of HPV52 or HPV58 in HPV33 multiple infections showed no significant association with the risk of cervical lesions, suggesting that their additional contribution to cervical lesion risk may be limited in the context of HPV33 co-infection. A large-scale meta-analysis by Chan et al. reported that the prevalence and attribution of HPV52 and HPV58 declined as lesion severity increased (Chan et al., 2014). Similarly, several recent clinicopathological studies found that women with HPV52 or HPV58 single infections had significantly lower rates of CIN3 and invasive cervical cancer than those with HPV16 infections (Lee et al., 2024). Epidemiological data also supported that (Ye et al., 2024). These findings collectively indicate that, although HPV52 and HPV58 are common in the general population, their contribution to malignant transformation appears to be limited, warranting further investigation into their biological behavior and regional variability. Meanwhile, there is no doubt that HPV16 continues to hold an unchallenged position as the most carcinogenic HPV genotype and may even overshadow the carcinogenic potential of other genotypes (Mirabello et al., 2018; Wu et al., 2019; Song et al., 2022).

Incidentally, HPV53 and HPV81 appeared to be protective factors within HPV33 dual infections in this study, which might be attributed to the limited number of infections observed, and further validation with a larger sample size was warranted. Moreover, most L1 detected sequences in this study belonged to the A1 sublineage, no matter in HPV33 single or multiple infection groups. As a result, no significant difference was observed in the related analysis. The limited number of sequences may be responsible for this. However, it is possible that insufficient selection pressure has not led to the accumulation of favorable mutations, which means that the HPV33 multi-infection variants may still establish a dominant position in the context of rich genetic diversity in the future.

Overall, it is the first study to provide a detailed analysis of the relationship between HPV33 co-infection patterns and cervical lesions. In addition to focusing on the number of co-infecting genotypes, we emphasized the important role of HPV16 in multiple infections through adequate analysis of the related risk among the various infection combinations. The clinical characteristics of enrolled patients were insufficient for a comprehensive analysis of factors associated with cervical lesions. Nonetheless, our findings underscore the need for genotype-specific evaluation, especially HPV33 multiple infections, in cervical cancer screening and prevention strategies. Compared with HPV16, HPV52, and HPV58, the relatively lower prevalence of HPV33 might obscure its substantial contribution to precancerous lesion development. Routine screening for HPV33 multiple infections should be incorporated into follow-up protocols, and further investigation is warranted. Our findings also provided new insights into the potential synergistic mechanisms, clinical implications, and future optimization of the vaccine defense strategy of HPV co-infections.

## 5 Conclusion

In this study, HPV33 co-infection was common and accounted for 51.86% of all the samples. Both the number and specific HPV genotypes are key factors that need to be considered in multiple infection analysis. Among HPV33 dual infections, those involving HPV52, HPV16, HPV58, or HPV31 were associated with an increased risk of cervical lesions. However, as the number of HPV33 co-infecting genotypes increased, only HPV16 retained a robust and independent association with the risk of cervical lesions, indicating a dominant oncogenic contribution relative to HPV52 and HPV58. In addition, the predominant sublineage of HPV33 in multiple infections remains A1. Future studies are needed to demonstrate these findings mechanistically, specifically regarding the impact of genotype-specific interactions on the prognosis of HPV infection, immune response, and the outcome of clinical treatment.

## Data Availability

The original contributions presented in the study are included in the article/Supplementary material, further inquiries can be directed to the corresponding authors.

## Supplementary material

Supplementary Figure S1 Flowchart of patient enrollment. **(A)** Flowchart of patient enrollment for the association of HPV33 co-infections with cervical lesions (Part 1). **(B)** Flowchart of patient enrollment for the association of HPV33 genetic diversity with susceptibility to multiple infections (Part 2).
