# Supplementary figures and images for: Association between multiple infection patterns of HPV33 and the risk of cervical carcinogenesis
Source: Front Microbiol. 2026 May 25;17:1787378. doi: 10.3389/fmicb.2026.1787378 (PMC13243004; doi:10.3389/fmicb.2026.1787378)

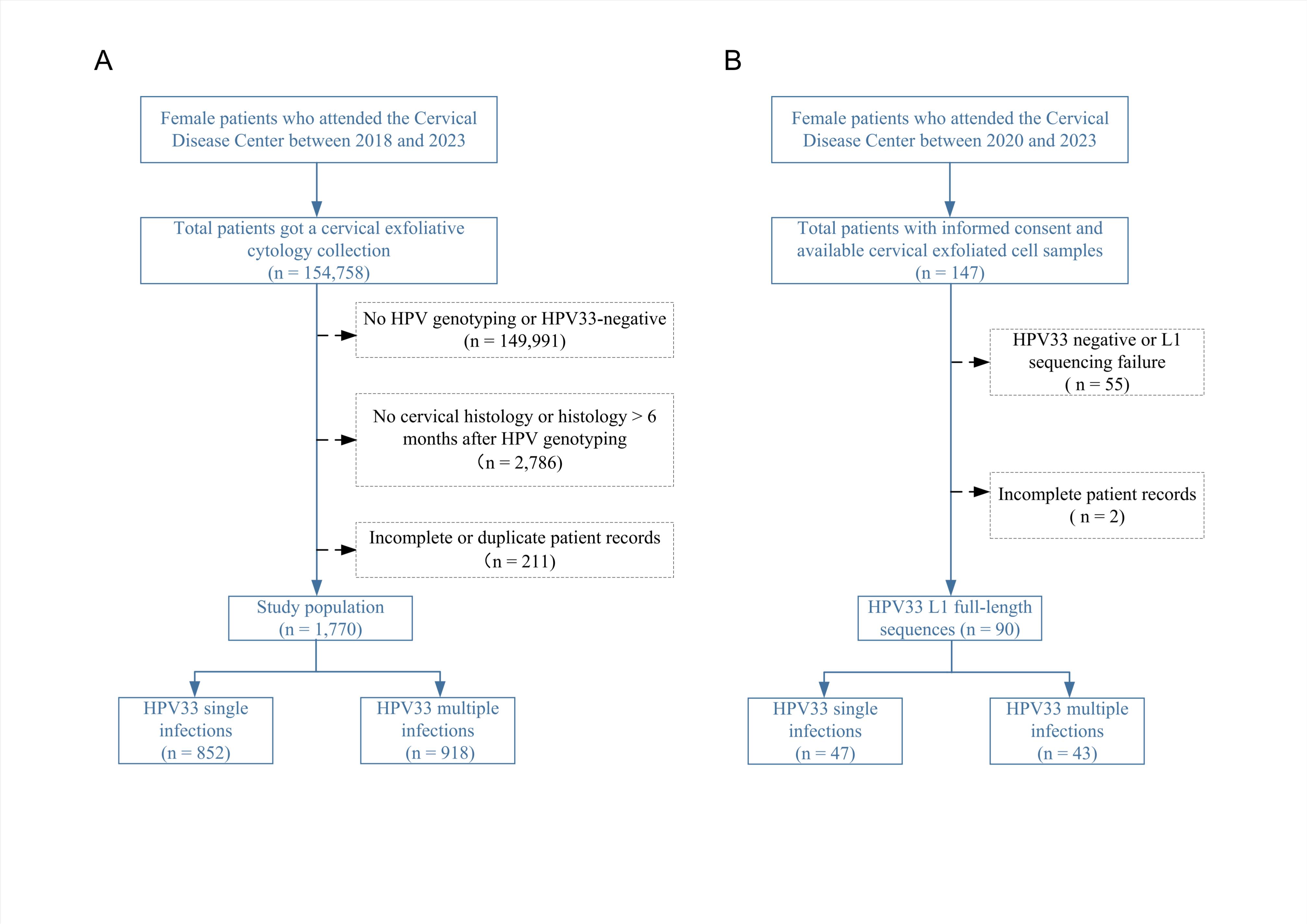

Supplement: Supplementary Figure S1 — Flowchart of patient enrollment. (A) Flowchart of patient enrollment for the association of HPV33 co-infections with cervical lesions (Part 1). (B) Flowchart of patient enrollment for the association of HPV33 genetic diversity with susceptibility to multiple infections (Part 2). [file Image_1.jpeg]
